# A simulation-based curriculum to introduce key teamwork principles to entering medical students

**DOI:** 10.1186/s12909-016-0808-9

**Published:** 2016-11-16

**Authors:** Arna Banerjee, Jason M. Slagle, Nathaniel D. Mercaldo, Ray Booker, Anne Miller, Daniel J. France, Lisa Rawn, Matthew B. Weinger

**Affiliations:** 1 Department of Anesthesiology, Vanderbilt University Medical Center, 1211 21st Avenue S, 526 MAB, Nashville, TN 37212 USA; 2 Department of Medical Education, Center for Experiential Learning and Assessment, Vanderbilt University Medical Center, Nashville, TN USA; 3 Center for Research and Innovation in Systems Safety (CRISS), Institute for Medicine and Public Health, Vanderbilt University Medical Center, Nashville, TN USA; 4 Department of Anesthesiology, Vanderbilt University Medical Center, Nashville, TN USA; 5 Department of Biostatistics, Vanderbilt University Medical Center, Nashville, TN USA; 6 School of Nursing, Vanderbilt University Medical Center, Nashville, TN USA; 7 Present Address: Clinical and Rehabilitation Sciences Research Group, Faculty of Health Sciences, University of Sydney, Sydney, Australia; 8 Present Address: Baptist Health Neurosurgery Arkansas, Little Rock, AR USA; 9 Health Services Research Division and the Geriatrics Research Education and Clinical Center, Veterans Affairs (VA) Tennessee Valley Healthcare System – Nashville Campus, Nashville, TN USA

**Keywords:** Teamwork, Communication, Simulation, Interpersonal skills, Curriculum development, Iterative curriculum design, Course evaluation, Standardized patients, Undergraduate medical education, Geriatrics education

## Abstract

**Background:**

Failures of teamwork and interpersonal communication have been cited as a major patient safety issue. Although healthcare is increasingly being provided in interdisciplinary teams, medical school curricula have traditionally not explicitly included the specific knowledge, skills, attitudes, and behaviors required to function effectively as part of such teams.

**Methods:**

As part of a new “Foundations” core course for beginning medical students that provided a two-week introduction to the most important themes in modern healthcare, a multidisciplinary team, in collaboration with the Center for Experiential Learning and Assessment, was asked to create an experiential introduction to teamwork and interpersonal communication. We designed and implemented a novel, all-day course to teach second-week medical students basic teamwork and interpersonal principles and skills using immersive simulation methods. Students’ anonymous comprehensive course evaluations were collected at the end of the day. Through four years of iterative refinement based on students’ course evaluations, faculty reflection, and debriefing, the course changed and matured.

**Results:**

Four hundred twenty evaluations were collected. Course evaluations were positive with almost all questions having means and medians greater than 5 out of 7 across all 4 years. Sequential year comparisons were of greatest interest for examining the effects of year-to-year curricular improvements. Differences were not detected among any of the course evaluation questions between 2007 and 2008 except that more students in 2008 felt that the course further developed their “Decision Making Abilities” (OR 1.69, 95% CI 1.07–2.67). With extensive changes to the syllabus and debriefer selection/assignment, concomitant improvements were observed in these aspects between 2008 and 2009 (OR = 2.11, 95% CI: 1.28–3.50). Substantive improvements in specific exercises also yielded significant improvements in the evaluations of those exercises.

**Conclusions:**

This curriculum could be valuable to other medical schools seeking to inculcate teamwork foundations in their medical school’s preclinical curricula. Moreover, this curriculum can be used to facilitate teamwork principles important to inter-disciplinary, as well as uni-disciplinary, collaboration.

**Electronic supplementary material:**

The online version of this article (doi:10.1186/s12909-016-0808-9) contains supplementary material, which is available to authorized users.

## Background

Failures of teamwork and interpersonal communication have been cited as a major patient safety issue [1–6]. The 1999 Institute of Medicine (IOM) report, *To Err is Human* [7], reported a very high rate of preventable medical errors associated with dysfunctional teamwork or failed communication. Such failures may be especially high in patients with multiple complex conditions, in emergencies, and during care transitions. The Joint Commission identified communication as a critical factor in more than 65% of reported sentinel events [8]. For example, Mazzocco and colleagues demonstrated that surgical teams that exhibited fewer team behaviors had worse patient outcomes [6].

**Table 1:** Lessons Learned

Lesson Learned	Experiential Evidence	Literature Evidence
Each student needs to be actively engaged in an exercise to get maximum individual and team value.	Changes in *Crisis on Flight 1974* after TWD 2007 significantly increased student evaluations.	[55–64]
Faculty need appreciable training as well.	Faculty facilitators and debriefers who missed the 2-h group training felt much less prepared to teach the course (even after one-on-one review of the syllabus with the course director).	[65–67]
The order of sequential exercises is important and should support scaffolding of desired KSA.	Reorganization of TWD 2008 to assure that all students had *Williams Medical Center* before *Ward Rounds* significantly improved student ratings of the former exercise.	[66, 68]
Simulation-based training, especially of large student cohorts, is very faculty and support staff intensive.	TWD 2010 required **10** faculty facilitators, **12** faculty debriefers, **8** educational support staff, and **32** standardized patients/passengers for 7.75 contact hours for 101 students.	[69, 70]
Student preparation, especially for role play exercises, is critical to student learning and satisfaction.	Student comments and ratings of handouts and the course were lower in TWD 2007 and 2008 before greater effort was made to emphasize weekend study of their roles.	[71, 72]
Iterative curricular design based on students’ course evaluations is effective at improving an integrated course.	Tables S2 and S3.	
A comprehensive teamwork training exercise can be delivered successfully to new medical students.	Tables S2 and S3.	

In 2002, another IOM report dealing with healthcare curricula highlighted the importance of teamwork and communication as a core competency for healthcare professionals [9]. Yet, formal teamwork training is still uncommon in healthcare [10–12].

Increasingly, patients are cared for by distributed multidisciplinary teams of specialized healthcare professionals. Yet, the training of new physicians tends to be uni-disciplinary, with few formal interactions with other healthcare providers until post-graduate training [13, 14]. To be effective team members, individuals must learn teamwork competencies. A team consists of a small number of people with complementary skills who are committed to a common purpose and perform goals for which they are mutually accountable [15]. A successful healthcare team must also maintain a common understanding of what is to be done and what constitutes success; make a commitment to achieving the team goals, technical competence (i.e., the collective knowledge and tools necessary to do the job), effective team communication (which requires shared information, trust, and respect), and effective coordination and collaboration; and develop an effective team culture [16].

The notion that communication and teamwork are essential to medical education is not new. Crisis Resource Management (CRM) techniques borrowed from aviation have been widely used to teach teamwork skills to healthcare providers, for example, in anesthesia [17] and emergency medicine [18]. The Agency for Healthcare Research and Quality (AHRQ) and the Department of Defense have collaboratively developed the Team Strategies and Tools to Enhance Performance and Patient Safety (TeamSTEPPS) tool intended to improve patient safety by training healthcare providers in interpersonal communication and other teamwork skills [19].

Traditionally, medical school curricula have not included instruction in the specific knowledge, skills, attitudes, and behaviors required to function as part of interdisciplinary care teams. The current literature on teamwork curricula for physicians is sparse and, despite high face validity, there is as yet no high quality evidence that teamwork training of medical students has long-term or patient care quality impact [20–23]. Charkraborti, et al. [24], in a 2008 systematic review, identified only 13 studies of teamwork training in medical school curricula. Most of these few initiatives were multidisciplinary, including medical trainees, nurses, social workers, physical and occupational therapists, administrators, and pharmacists. All employed active learning methods, including critical incident simulations, role-playing, case-based scenarios, and actual patient encounters, but none used standardized patients or simulated clinicians (i.e., actors trained to realistically portray clinicians). Three studies described a focus on non-medical teambuilding exercises. Most of the curricula incorporated trainee feedback; seven used formal debriefing sessions and five used facilitated reflection. None of these studies were randomized or controlled or used previously validated assessment tools.

Nevertheless, in light of increasing evidence that care teams with better teamwork skills deliver better care [25–30], teamwork principles are being introduced into more medical school curricula [14, 31, 32]. There are no guidelines about the timing, extent, quantity, or assessment of this education. When should these important topics be introduced to undergraduate medical students? One view is that they should be introduced early in the professional career, before the inculcation of negative interpersonal attitudes (e.g., negative role modeling in the clinical years) that may be more resistant to change [33]. Others assert that teamwork education will only be effective when introduced to trainees who already have adequate clinical knowledge and experience [34].

Regardless, many educators agree there is a need to develop better methods to teach teamwork and communication skills [24, 31, 35]. Adults learn best through active participation, self-reflection, and the use of multimodal learning strategies (i.e., combining visual, auditory, and kinesthetic reinforcement) [36]. Experiential learning is an effective approach to facilitate adult education [37]. The most powerful learning experiences are ones where the learner is immersed in an event that simulates the real world. Such situations (when accompanied by structured debriefing of the experience) not only provide active learning of the desired knowledge, skills, and attitudes (KSA) but may increase the likelihood that the learners will be able to apply these KSA when confronted with similar real-world situations (i.e., successful transfer of training) [17, 38–43].

### Project goals

In 2007, Vanderbilt University School of Medicine modified their undergraduate medical education curriculum to include a two-week *Foundations of the Profession* course, designed to introduce new medical students to the most important themes in modern healthcare. As part of this curriculum, we created a day-long experiential course on teamwork and interpersonal communication. *Teamwork Day* (TWD) was offered to all entering students for five consecutive years on Day 6 of medical school. The goal of TWD was to introduce the students to the core knowledge, skills, and attitudes required to be effective leaders and members of healthcare teams. In this paper, we describe our first four years’ experience developing, implementing, evaluating, and iteratively refining this unique, simulation-based medical curriculum.

## Methods

This study was granted an exemption from requiring ethics approval by the Vanderbilt University Institutional Review Board (IRB#141602).

### Curriculum development

The Teamwork Day Curriculum Committee consisted of a broad multidisciplinary coalition of faculty from numerous clinical disciplines (anesthesiology, emergency medicine, internal medicine, nursing, pediatrics, and surgery), medical educators, human factors engineers, simulationists, and organizational behaviorists. This group of faculty defined the core educational objectives based on a thorough review and synthesis of the literature [44–52]. The broad goals of TWD were to introduce new medical students to: 1) What teamwork is and why it is important to them and to healthcare; 2) The essential characteristics of teams; 3) The essential dimensions of teamwork; 4) What effective teams do (and don’t do); and 5) The core KSA of teamwork that they will need throughout their medical career. This information was presented to the students in their preparatory binder and summarized at the start of the day in a succinct presentation by the faculty teaching the course. An additional curricular design goal was to make the exercises professionally relevant and engaging to new medical students, who were largely devoid of clinical knowledge or experience.

Based on the overall curricular goals, individual learning objectives, and the logistical constraints of delivering a 1-day course to 100 students, the faculty met repetitively to develop the teamwork exercises using an iterative user-centered design approach [53]. Six 75-minute learning sessions were developed to address the course objectives. Each session included at least one participatory exercise and faculty-facilitated peer debriefing. The exercises incorporated a variety of interactive simulation modalities, including role-playing; computer-controlled manikins; simulated team members; and standardized patients. Except for the brief introduction, *formal didactics were avoided*. Each learning session was pilot-tested and refined using a subset of the faculty as mock students and, then, two sessions of high-school students.

A comprehensive syllabus was developed that included an introductory treatise about TWD and teamwork principles, general exercise descriptions, individualized roles and instructions (as needed), and preparatory reading. The syllabus, placed in a three-ring binder, was customized for each student based on his or her group and exercise role assignments. Since TWD occurred on the second Monday of the first year of medical school, the syllabi were distributed to students the Friday morning beforehand. Beginning with TWD 2008, the course director made a brief presentation that Friday morning in which the importance of reading the syllabus and learning one’s roles over the weekend was emphasized.

### Simulation exercises

The six teamwork exercises created for TWD 2007 are described briefly below and in Additional file 1: Table S1. More detail about each exercise and its instructional materials can be obtained from https://medschool.vanderbilt.edu/cela/stp-courses.

#### Getting to know Who?

This is a simple teambuilding exercise modified from one developed in 1996 at Stanford University. The educational objectives include: basic teambuilding, trust, team decision-making, and dealing with conflict. In this exercise, each team member writes down, on two 3” × 5” cards, two little known facts about him- or herself. The items chosen should shed light on a personality trait or other personal aspect of the person’s life without immediately revealing the identity of the individual. The students are informed that more unusual or controversial items generate the best discussions. The faculty facilitators provide examples of such items (e.g., “I was thrown in jail during a spring break vacation” or “I gave a child up for adoption”). The cards are collected by the facilitator who then chooses one and reads it aloud to the group. The team is then asked to determine who wrote the card. Team members may choose, to vote or to try to reach consensus. Once a student is identified as the author, he or she has the option of either keeping quiet, trying to mislead the group, or admitting to being the author. The faculty may ask the student to express how it felt to be so identified (e.g., as a “male chauvinist,” “total nerd in high school,” etc.). Typically, the team goes through 4 to 8 of the cards before moving on to the second exercise of this initial session.

#### Colour Blind™

Colour Blind™ (RSVP Design, Johnstone, UK) was originally developed for air traffic control cadets. The educational objectives include sense-making, listening, leadership styles, information management, and the importance of cross checking and feedback. Team members all wear blindfolds to ensure total dependence upon the quality of their verbal communication. The group works together to gather information that will allow them to solve a puzzle. They must ascertain, with minimal input from the instructor, which two-colored and abnormally shaped pieces are missing from a set of 30 pieces (consisting of 6 shapes and 5 colors). The debriefing commonly addresses issues of leadership, followership, and communication.

#### Mission to Burundi™

The objectives of this locally developed exercise include understanding the individual contributions to a team decision and the relative influence of individuals who may or may not have relevant expertise. Students are told that they are part of a medical team responding to a humanitarian disaster in a small African nation. Students are not assigned any roles but asked to be themselves for this exercise. Each participant is asked to prioritize individually the supplies and equipment that must accompany the team within the limited space on their airplane. The team then convenes to discuss and agree upon a final prioritized list. Both individual and group lists are then compared to a list previously created by a consensus of faculty experts in global health and humanitarian medical relief missions. Individual and team scores are based on concordance with the expert rankings. During the debriefing, team scores are compared across several teams, and facilitated discussion focuses on how team decisions occurred. Generally, teams do better than individuals. If an individual’s score is better than her team’s, it suggests that she was not adequately influential (or the team was not adequately receptive to her input) during the team decision process.

#### Williams Medical Center^TM^

This session introduces the students to basic negotiation principles through the use of a multiparty negotiation exercise provided by the Program on Negotiation at Harvard Law School (Cambridge, MA). The objective of this exercise is to introduce students to basic negotiation principles and skills. Each student is assigned a specific role on the Pharmacy and Therapeutics (P&T) Committee of Williams Medical Center, a 1,000-bed, university-affiliated, non-profit facility located in a large metropolitan area. The students are charged with making a decision (as the committee) about how to regulate physicians’ ability to prescribe high-risk specialty drugs. There have been two large malpractice suits in the past year related to non-specialist physicians’ prescribing medications that led to adverse drug events. Each P&T Committee member is assigned a strong opening position from which he or she must retreat if the committee is to reach a decision on which a majority of the committee members will agree. Students must discern who their allies and antagonists are on each position, convince others of the strength of their positions, and reach a negotiated settlement. Points are awarded to each individual for each agreed upon aspect of the final decision (assuming agreement was reached). The facilitated debriefing addresses both the processes and outcomes of this structured negotiation problem.

#### Ward rounds at Jefferson County Medical Center™

This is a locally developed, complex, multiparty negotiation role-play exercise that includes an elderly standardized patient (SP). The educational objectives are to develop negotiation and influence skills, clinician-patient interactions, and appropriate clinician-clinician interactions in the presence of a patient. The students are assigned roles on a clinical care team making hospital discharge decisions about a geriatric patient. The inpatient care team, which consists of a charge nurse, physical therapist, hospitalist physician, orthopedic surgeon, and social worker, must decide how best to manage five clinical or social aspects of this patient’s discharge process – the location to which the patient should be discharged, how to manage the patient’s invalid spouse, the appropriate rehabilitation therapy regimen, how to manage the patient’s insulin-dependent diabetes, and how to manage the patient’s refractory atrial fibrillation. The team first meets together and tries to reach consensus on the five inter-related decisions. The geriatric SP is then wheeled into the room.

The SP is trained to assume negotiation positions that are designed to be contrary to what the students collectively are likely to decide for the patient in his or her absence. The exercise has two equilibria (i.e., balance of collective decisions of the involved parties on the five decision elements) – one when the five students negotiate together without the patient and a different one when the SP joins the negotiation. The students must present their decision to the patient and then try to negotiate with the patient, who has different preferences. The SP is trained to be appropriately but incompletely influenced to retreat from his/her inherent preferences. This is the students’ first medical school opportunity to interact with a patient, albeit a standardized one, and the realization that patients cannot be excluded from decisions affecting them is a powerful experience.

#### Crisis on flight 1974™

The educational objectives of this locally developed role-play simulation exercise are to introduce the students to principles of crisis resource management [17, 18]. The students are given roles as passengers or flight attendants on a transcontinental flight that goes awry (based on an actual event). Students’ roles and provided materials contain information necessary to manage the event. Three other passengers are actors who have distractor roles (e.g., a woman with a crying baby, a passenger with severe ear pain). In 2007, the “patient” role was played by a computer-controlled manikin (Laerdal SimMan™). After the passengers settle in and the captain announces the need for seatbelts due to inclement conditions, the simulator-passenger has a seizure. The students must manage the initial bedlam, ascertain that the patient is an insulin-dependent diabetic, measure the passenger’s blood sugar (which is very low) using a glucometer, administer an oral sugar-containing product, and then make a triage decision (i.e., flight diversion) when the patient doesn’t completely return to normal. Debriefing focuses on situation awareness, resource allocation, management of uncertainty and limited resources, dynamic leadership, and other CRM principles.

### Course logistics and evaluation

Upon completion of the daylong course, every student was asked to evaluate each session, their team facilitator, and overall course attributes. The evaluation form used 7-point scales, with 1 being the lowest (worst) and 7 being the highest (best) score. We tracked the scores annually and iteratively adjusted course structure and content to improve the course.

The two main goals of the analysis of these evaluations were to describe students’ perceptions of the course, and of the simulation exercises, over the four-year period and to determine if there were any temporal changes or changes between exercises as the course was iteratively refined. The distribution of each evaluation item was summarized as the mean and standard deviation and the median and interquartile range (IQR, 75th – 25th quantile). Two ordinal logistic regression models were created to quantify the association between the scores and the course evaluations and between the scores and the simulation exercises. Each model contained an interaction between the evaluation (course or simulation exercise) and year, while the model pertaining to the simulation exercise also contained a term characterizing the order in which the exercise was given. All adjustment terms were modeled as a series of indicators. Robust, or ‘sandwich’, standard errors were calculated to account for the correlations within the data due to student group and instructor assignments. Estimates summarizing key parameter combinations (e.g., *Getting to Know Who* during 2008 vs *Getting to Know Who* during 2007, *Getting to Know Who* during 2009 versus *Colour Blind* during 2009), along with their associated 95% confidence intervals (CI), were calculated and then exponentiated to produce odds ratios (OR) and their CIs. P values from the Wald test (comparing OR = 1 vs OR ≠ 1) were also reported. All analyses were performed in R [54].

**Fig. 1:**
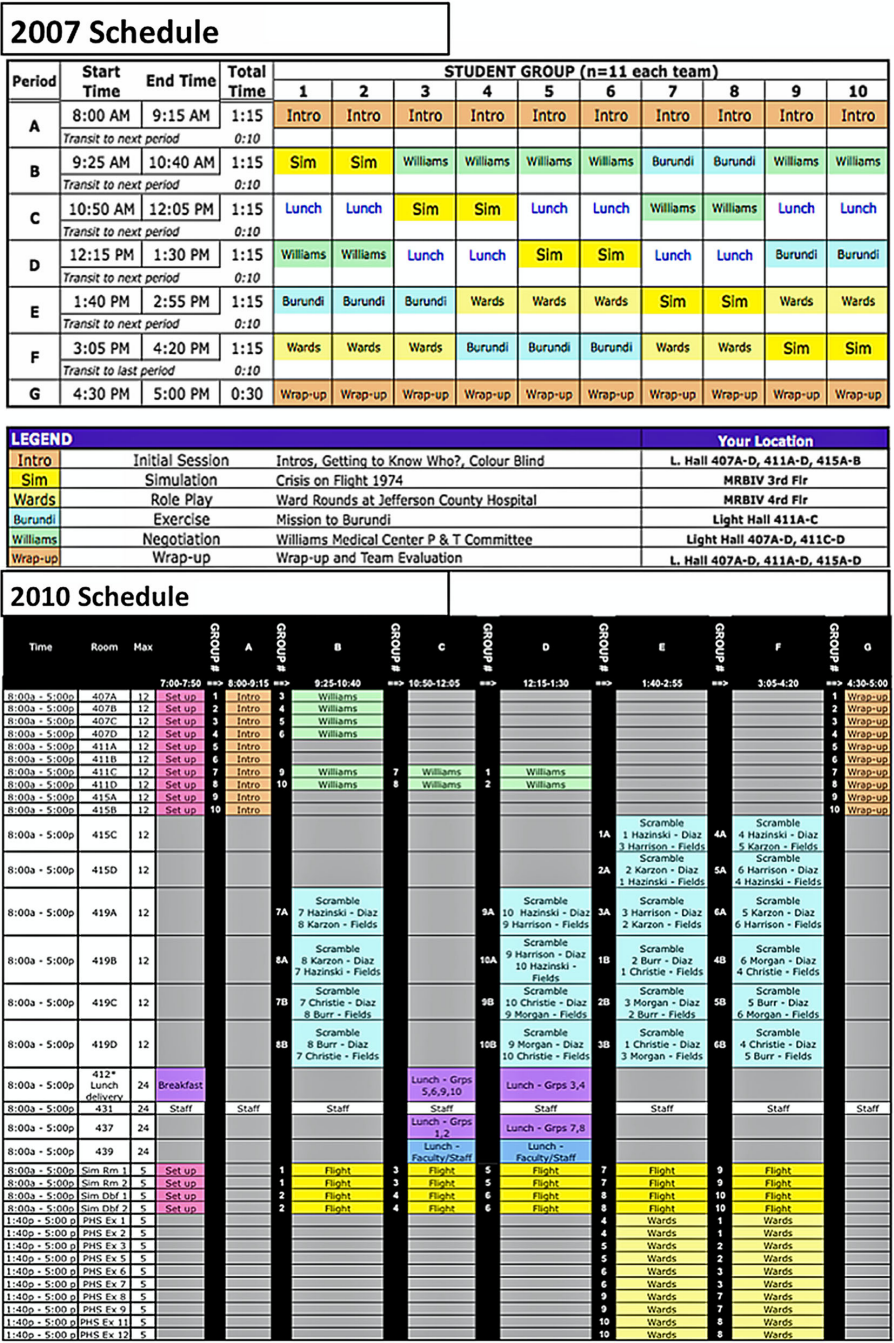
Teamwork day schedules for 2007 and 2010

We faced some logistical challenges especially in the first year. Providing a complicated, highly interactive course to an entire medical school class broken down into 10 teams (and often further into groups of 5 to 6) was very difficult. Because of limited capacity to conduct some exercises (*Ward Rounds at Jefferson County Medical Center* and *Crisis on Flight 1974*), these exercises had to be offered in different orders to different student teams (see Fig. 1). Other challenges included getting students from one session to another distributed across two adjacent buildings and two floors, as well as orchestrating 18 faculty and a dozen staff throughout the day.

## Results and iterative improvement

### 2007 Teamwork day

#### Implementation

All faculty underwent a formal two-hour course briefing. Both the faculty and the students received the course syllabus in advance. The 103 medical students were grouped into teams of 10 and assigned a faculty member for the day. Each student participated in all six sessions, each of which lasted 75-min, plus a lunch period. Additional trained faculty supported group de-briefing of the exercises.

#### Review of the 2007 course

Several weeks after the course, the Curriculum Committee met to carefully review and discuss the collated numeric evaluations, narrative comments, and personal observations. Overall, the course was extremely well received by the students (see Additional file 2: Table S2). The syllabus received the lowest average ratings. Multiple student and faculty suggestions were implemented for its improvement in the 2008 course. Student ratings were lower than expected for two exercises, *Williams Medical Center* and *Crisis on Flight 1974*. For the 2008 course, we improved the facilitator instructional materials for *Williams Medical Center*. Review of the data also suggested that students who did *Williams Medical Center* after they had completed *Ward Rounds at Jefferson County Medical Center* gave the former a lower score and complained that the two were redundant in content. Therefore, for 2008, we doubled the number of available geriatric patients for *Ward Rounds at Jefferson County Medical Center* so that the students could always do *Williams Medical Center* first. The evaluations also suggested that the *Getting to Know Who* exercise seemed to work best in groups where members already know each other somewhat and have an initial level of comfort.

Upon review of the comments and ratings for *Crisis on Flight 1974*, the faculty determined that there were not enough actively participating roles when an entire team (10–11 students) was involved in a single airplane cabin scenario. In the 2007 version, there were 7 student interactive roles and 3 non-student roles (SimMan^TM^, a standardized actor sitting next to the manikin, and the captain of the airplane [voice from the control room]). Four of the students had strictly observational roles. The use of realistic (i.e., high-back) airplane seats made it difficult for most of the students to see what was happening. Therefore, for the 2008 course, we changed the airplane seats and, more importantly, doubled the number of simulated airplane cabins (from two to four) that could be run concurrently so that only 5–6 students were in each scenario and all had the opportunity to participate in managing the crisis. In addition, we converted the seizing hypoglycemic passenger role from a manikin to a human actor. This required the design of a realistic simulated fingertip containing a red-colored low-glucose test solution so that the students could check the patient’s blood sugar with a commercial home glucometer (in the seizing patient’s carry-on luggage) to make the hypoglycemia diagnosis.

### Teamwork day 2008

The changes previously described were implemented in the 2008 course. Several new faculty were engaged to facilitate student teams. While the syllabus and facilitator de-briefing guide were re-written, and all faculty received pre-course training, a few of the new facilitators received less favorable scores than did those who had participated the previous year. Another change was the allocation of students to groups. In the first year of TWD, we kept the students in the same groups they had been in from day one of medical school orientation (2 weeks’ time together before the course, including orientation week). As a result, they actually already knew their group peers reasonably well. The TWD Curriculum Committee felt that putting students together who did not know each other quite as well would enhance their learning opportunities.

There were significant improvements in the scores between 2007 and 2008 for *Crisis on Flight 1974* (OR = 3.92, 95% CI: 2.24–6.86) and *Williams Medical Center* (OR = 1.72, 95% CI: 1.02–2.89, Additional file 2: Table S2). The students’ ratings of *Mission to Burundi* remained lower than those of the other participatory exercises (Additional file 3: Table S3). Narrative comments and instructor observations noted that this exercise was often completed in less than the 75 min allotted.

### Teamwork day 2009

The learning objective used to create the exercise *Mission to Burundi* was that team decisions can be more effective than individual decisions. The curriculum team felt that this objective was already being met by the other sessions. This, in addition to its lower ratings, led the curriculum team to replace *Mission to Burundi* with a new exercise for the 2009 course, which was ultimately named *Pediatric Surgery Scramble* (see below and the latter part of Table S1 in Additional file 1). Another change implemented for TWD 2009 was to mix up the student role assignments in the exercises involving only 5 to 6 students (number of students on a full team = 10 to 11). In 2008, some students complained that they ended up interacting with the same students in several different exercises and that this diminished their ability to try out different interaction styles. We also further revised the syllabus and reading assignments.

The curriculum team decided that the core educational objective of the new exercise would be to introduce students to distributed cognition and coordination. While some healthcare teams routinely work in close physical proximity, many others work together while separated across both space and time. In co-located teams, critical task and other information are often simultaneously available to many team members who can modify their decisions and actions based on immediate (visual or verbal) feedback from their colleagues. In contrast, in distributed teams, deliberate communication and collaboration practices are required to assure effective team coordination. The design of the new exercise was also based on the educational objective to introduce students to the roles of non-physician healthcare providers and to the nature of inpatient medical care typically performed by interns and senior clerks. *Pediatric Surgery Scramble* evolved over several months of effort into an exercise in which student teams were charged with getting two pediatric patients (played by teddy bears) ready for urgent surgery by orchestrating, for each patient, a series of required pre-operative consents, tests, consults, and procedures. The students needed to determine and orchestrate an efficient way to accomplish all of the tasks within a limited time frame. The team starts on the ward with their two patients but then must order tests, transport the patient to radiology and cardiology, transport specimens to the clinical laboratory, find a Spanish translator for surgical consent from a parent of one of the patients, etc. We pilot tested *Scramble* twice, first with the faculty facilitators (as part of their pre-course training) and then with a group of high school students.

The first offering of *Pediatric Surgery Scramble* was challenging. The curriculum team discovered a number of short-comings, including unclear instructional materials, insufficient clinical support staff that created excessive bottle-necks for the students (e.g., only one Spanish translator, only one medical receptionist, and one patient admission card stamping machine), and too many tasks. Some of the student groups became unduly frustrated by their failure to accomplish even half of the assigned tasks.

### Teamwork day 2010

During faculty debriefing of TWD 2009, the curriculum team noted that the very first exercise of the day, *Getting to Know Who*, was the only one in which student evaluations were decreasing year-to-year (Additional file 3: Table S3). It was therefore decided to replace this exercise with two new ones (see bottom of Additional file 1: Table S1). We created an alternate introductory ice-breaker (the *Name Game*) in which each student in a group would reveal their *full* name and why their parents gave them those names (which brought up interesting cultural discussions given our multicultural medical school classes), where they were from, where they went to school, and one thing that they “really hated.” Other students were encouraged to ask questions for clarification. After all of the students had a chance to present and answer questions, the team did a second brief physical exercise called *Get to your Spot*. In this exercise, students stand in a circle and hold hands. The students are instructed to pick a spot on the floor that is their “home,” and the goal is to “get to their spot.” They are asked not to talk and not to let go of each other’s hands. The first time, the students are told that the first one to get to his or her spot wins. There is a lot of tugging and pulling, and the exercise is typically over in seconds. The exercise is then repeated with all the same instructions except “when everyone gets to their spot the team wins.” The behavior is quite different; after a bit more time and a fair bit of non-verbal communication (e.g., hand tugging), everyone has touched their spots.

Based on the prior year’s experience, we made a number of changes to *Pediatric Surgery Scramble*. We reduced the number of required tasks per patient, clarified instructions, added extra SPs playing the various roles, including parents, medical receptionist, and Spanish translator. We also clarified and standardized the responses of the confederates playing the roles of the radiology technician, cardiology receptionist, clinical lab technician, and ward nurses.

The students did not rate their group facilitators as highly in 2010 as in the previous year (Additional file 2: Table S2). While this might be explained by the fact that some of the best facilitators from 2009 were not available to teach in 2010 or a modest decrease in faculty preparatory training, it may also represent sampling error or regression toward the mean. Note that over the four years, the range of means for this question (#4) was only between 6.4 and 6.7 (out of 7). Nevertheless, two facilitators with lower ratings were dropped from teaching in TWD 2011. Figure 1 shows the team and room assignments for TWD 2010, which exemplifies the results of our logistical refinements, made over the four years.

### General findings

A total of 420 evaluations were collected (2007: *n* = 103, 2008: *n* = 105, 2009: *n* = 111, 2010: *n* = 101). For a first-time offering in 2009, *Pediatric Surgery Scramble* received very good quantitative and narrative evaluations (notably better than those received by the *Mission to Burundi* exercise that it replaced). Overall, TWD course evaluations were quite positive, with almost all questions with means and medians greater than 5 out of 7 (best possible) across all four years (see Additional file 2: Table S2). Sequential year comparisons (e.g., 2008 vs 2007) were of greatest interest for examining the effects of attempted year-to-year curricular improvements. Differences were not detected among any of the course evaluation questions between 2007 and 2008 except that more students in 2008 felt that the course further developed their “Decision Making Abilities” (OR 1.69, 95% CI 1.07–2.67). With extensive changes to the syllabus and debriefer selection/assignment, concomitant improvements were observed in these aspects between 2008 and 2009 (OR = 2.11, 95% CI: 1.28–3.50). Substantive improvements in specific exercises also yielded significant improvements in the evaluations of those exercises (2007 versus 2008) – *Crisis on Flight 1974* (OR = 3.92, 95% CI: 2.24–6.86) and *Williams Medical Center* (OR = 1.72, 95% CI: 1.02–2.89). Similar interventions for *Pediatric Surgery Scramble* after its first year did not yield comparable improvements in students’ ratings, suggesting a need for further changes.

The *Ward Rounds* exercise tended to have the highest evaluation scores all 4 years (nearly all ORs > 1 and CIs excluding 1), followed by *Colour Blind*. A clear pattern was not observed among the remaining exercises (Additional file 3: Table S3 shows all comparisons between the exercises over time).

## Discussion

This paper describes an innovative and successful Teamwork Day curriculum that can be implemented in any medical school with a simulation program. We elucidate the four-year evolution and development of this one-day experiential learning program for entering medical students. As important, the findings show how iterative curricular refinement over a several year period selectively improves curricular elements, at least as evidenced by students’ perceptions of learning. Finally, from our experience, we have distilled a number of lessons (Table 1 [55–72]) for others trying to implement teamwork training for medical students.

Some of the key milestones in this process included deciding the program format, choosing activities, developing activities where none existed, and responding to student feedback. This education experience focused on the knowledge-, skill-, and attitude-based competencies [31] necessary for effective teams, but did so using an experiential rather than a didactic learning approach.

From the outset, the TWD Curriculum Team was committed to a deeply engaging, experiential learning approach in the belief that a strong, early impact would have longer-lasting effects. A design goal was to deliver to these nascent physicians a powerful “dose” [73] of teamwork KSA using state-of-the-art simulation methods. The more salient the experiences, the longer the learning retention lasted – years later we still hear from former students who tell us this was one of the highlights of medical school and who can recall relevant learning points and other details from the exercises. We were also committed to developing high-fidelity scenarios of situations that students would be likely to encounter in actual professional practice. These commitments strongly influenced which activities we chose to include in the curriculum. Some activities (e.g., *Colour Blind*, *Getting to Know Who*, *Williams Medical Center*) were readily available. These activities in particular were aimed at highlighting specific team-related skills (e.g., communication in *Colour Blind*; interpersonal assumptions and individual differences in *Getting to Know Who* and *Name Game*) and thus provided a basis on which to build the more sophisticated healthcare-specific exercises. *Mission to Burundi* and *Crisis on Flight 1974* extended communication skills and emphasized the importance of information sharing and the value of team decision-making in hypothetical and ‘actual’ situations. *Williams Medical Center* introduced conflict resolution and the politics of team interaction. Then, *Ward Rounds at Jefferson County Medical Center* harnessed this learning and allowed students to practice their negotiation skills, first together, and then in a complex situation of conflict between a multidisciplinary clinical team ward team members and the standardized patient. In 2009, *Pediatric Surgery Scramble* introduced the concept of distributed teams and the importance of distributed cognition, situation awareness, and team coordination [74, 75].

The simulation literature suggests that immediate feedback and reflection during a post-simulation debriefing may be the most important feature of this type of education [38, 40, 42, 43, 76]. Humans learn best when they learn through active participation [77, 78]. Simulation fulfills this need and at the same time allows subsequent analysis and reflection on the experience and facilitates incorporation of behavioral changes into personal practice. Reflective debriefing after each of our sessions was critical to the student’s learning experience. During debriefings, the students challenged their teams’ effectiveness, identified opportunities for improvement, and explored the learning’s relevance to past and future personal experiences. Faculty facilitators guided this reflective process and helped learners appraise their own and their team’s performance.

## Conclusions

In summary, we created a novel, highly experiential teamwork-training course to teach early first-year medical students basic teamwork and interpersonal principles and skills using state-of-the-art simulation methods. Through four years of iterative refinement, the course matured and was highly regarded by students and School of Medicine faculty. Some, or all, of this curriculum could be valuable to many other medical schools seeking to inculcate teamwork foundations in their programs. Moreover, this curriculum could be used to facilitate teamwork principles important to inter-disciplinary, as well as uni-disciplinary, collaboration, an avenue for future development by our team.

## Additional files

Additional file 1: Table S1. Exercises Used in Teamwork Day 2007 and 2008. (PDF 203 kb)
Additional file 2: Table S2. Teamwork Day Course and Exercise Evaluations Across the Years. (PDF 259 kb)
Additional file 3: Table S3. Yearly Comparison of Exercises (*P* values). (PDF 277 kb)

## Abbreviations

AHRQ: Agency for healthcare research and quality
CI: Confidence interval
CRM: Crisis resource management
IOM: Institute of medicine
IQR: Interquartile range
IRB: Institutional review board
KSA: Knowledge, skills, and attitudes
OR: Odds ratio
SD: Standard deviation
TeamSTEPPS: Team strategies and tools to enhance performance and patient safety
TWD: Teamwork day
USMLE: United States Medical Licensing Examination
