# Supplementary material for: A simulation-based curriculum to introduce key teamwork principles to entering medical students
Source: BMC Med Educ. 2016 Nov 16;16:295. doi: 10.1186/s12909-016-0808-9 (PMC5112730; doi:10.1186/s12909-016-0808-9)
Supplement: Additional file 1: Table S1. — Exercises Used in Teamwork Day 2007 and 2008. (PDF 203 kb) [file 12909_2016_808_MOESM1_ESM.pdf]

Table S1. Exercises Used in Teamwork Day 2007 and 2008

Exercise	Source	Teaching Method	Key Learning Objectives
Getting to Know Who?	M. B. Weinger and Stanford University School of Business (Palo Alto, CA)	Group discussion	This exercise was designed to facilitate the development of a non-judgmental atmosphere in the group. It will also allow individuals in the early stages of team development to:<br>• Gain an appreciation of group diversity.<br>• Practice decision making in a low-risk setting.<br>• Deal with conflict.
Colour Blind™	RSVP Design (Johnstone, Scotland, UK)	Team cognitive exercise	This exercise was designed to:<br>• Refine skills that will help ensure common understanding (sense making) between team members.<br>• Emphasize the importance of checking and feedback.<br>• Demonstrate how communication can break down in teams, and to enable teams to identify and rectify such communication failures.
Mission to Burundi	J. Slagle and Vanderbilt University School of Medicine (Nashville, TN)	Role play and decision evaluation	This role play exercise was designed to:<br>• Emphasize the factors that improve or impede team performance.<br>• Highlight relative contributions of individual

			team members versus the collective wisdom of the team as a whole.<br>• Explore the concept of "Groupthink" (social pressures to agree).
Williams Medical Center ™	The Harvard Negotiation Project™ (Cambridge, MA)	Multiparty negotiation with role play	This role-play exercise teaches the students basic multiparty negotiation skills including:<br>• Evaluate options and positions before and then during the bargaining process.<br>• Importance of separating personalities from the problems that must be resolved.<br>• Communicate effectively in an adversarial situation.<br>• Introduction to contingent agreements and post-settlement settlements.
Ward Rounds at Jefferson Hospital	M. B. Weinger and Vanderbilt University School of Medicine (Nashville, TN)	Role play with a Standardized Patient	This role-play exercise teaches the students more advanced negotiation and patient-interaction skills including:<br>• Understand the importance of and methods for team resolution of conflict situations.<br>• Understand and overcome barriers to effective communication.<br>• Effective information transfer (especially listening) - obtaining input and information from everyone on the team and from the patient.

			• Importance of interpersonal and cultural differences, and their influence on team decision-making.<br>• Integrating patient preferences into care decisions especially when they are in conflict with the providers' preferences.
Crisis on Flight 1974	R. Booker and Vanderbilt University School of Medicine (Nashville, TN)	Role play with high fidelity simulator (SimMan™) and simulated passengers (actors) in 2007. Only simulated passengers were used in 2008 and thereafter.	This exercise introduces students to the principles of crisis resource management (CRM) including:<br>• Recognition and assumption of team roles (leaders and followers).<br>• Team communication skills especially closing the loop and cross-checking.<br>• Resource identification and management including calling for help, getting what you need.<br>• Situation Assessment including dynamic changes in an open mind.[5]
**New Exercise for Teamwork Day 2009**			
Pediatric Surgery Scramble	Vanderbilt University School of Medicine	Team exercise with Simulated Clinicians	This exercise was designed to get students to:<br>• Appreciate challenges inherent in complex interdisciplinary health care delivery.<br>• Provide an introduction to the life of an inpatient house staff physician or third year clerk.

			• Develop and maintain effective goal-oriented team coordination.<br>• Develop strategies to maintain cohesive distributed teamwork.<br>• Learn preparation, planning, direction and execution of the team goals and objectives.<br>• Foster team assessment and provide process feedback related to team progress.
**New Exercises for Teamwork Day 2010**			
Name Game	M. Weinger and Vanderbilt University School of Medicine (Nashville, TN)	Group discussion	This exercise was designed to replace "Getting to Know Who" and facilitate the development of a non-judgmental atmosphere in the group. It also allows individuals in the early stages of team development to:<br>• Gain an appreciation of group diversity.<br>• Engage in team conversation and learning.
Get to Your Spot	Origin unknown	Team physical exercise	This simple exercise was designed to:<br>• Demonstrate the difference between pursuing team goals versus individual goal.
