# Supplementary material for: A simulation-based curriculum to introduce key teamwork principles to entering medical students
Source: BMC Med Educ. 2016 Nov 16;16:295. doi: 10.1186/s12909-016-0808-9 (PMC5112730; doi:10.1186/s12909-016-0808-9)
Supplement: Additional file 2: Table S2. — Teamwork Day Course and Exercise Evaluations Across the Years. (PDF 259 kb) [file 12909_2016_808_MOESM2_ESM.pdf]

Table S2. Teamwork Day Course and Exercise Evaluations Across the Years

		Count / Missing	Mean ± (SD)	Median (IQR)	Signif. Change 2007 to 2008?	2008 vs. 2007 (ref)	Signif. Change 2008 to 2009?	2009 vs. 2008 (ref)	Signif. Change 2009 to 2010?	2010 vs. 2009 (ref)
						OR (95% CI) P		OR (95% CI) P		OR (95% CI) P
**Overall Course Evaluation Questions**										
1. The course developed skills and techniques required to effectively collaborate with other team members	2007	103 / 0	6.0 ± (0.8)	6.0 (2.0)		1.19 (0.74–1.91) 0.48		1.30 (0.82–2.05) 0.27		1.04 (0.67 – 1.60) 0.87
	2008	100 / 0	6.0 ± (1.0)	6.0 (1.0)						
	2009	110 / 1	6.2 ± (0.8)	6.0 (1.0)						
	2010	101 /0	6.1 ± (0.9)	6.0 (1.0)						
2. Handouts and other distributed materials were valuable supplements to the course	2007	103 / 0	5.4 ± (1.1)	5.0 (1.0)	Revised syllabus and changed some readings	1.09 (0.65–1.81) 0.75	Major revision of syllabus and new readings	2.11 (1.28–3.50) **0.004 ***		0.85 (0.50 – 1.42) 0.53
	2008	100 / 0	5.4 ± (1.1)	5.5 (1.0)						
	2009	111 / 0	5.8 ± (1.1)	6.0 (2.0)						
	2010	100 / 1	5.8 ± (1.0)	6.0 (1.2)						
3. The course resulted	2007	103 / 0	5.6 ± (0.9)	6.0 (1.0)	Improved	1.69		1.23		0.81

in further development of my decision-making abilities	2008	99 / 1	5.8 ± (1.0)	6.0 (1.0)	several exercises	(1.07–2.67) **0.026 ***		(0.75–2.03) 0.41		(0.47 – 1.41) 0.46
	2009	111 / 0	5.9 ± (1.1)	6.0 (2.0)						
	2010	101 / 0	5.8 ± (1.1)	6.0 (2.0)						
4. Team facilitator was effective	2007	101 / 2	6.6 ± (0.6)	7.0 (1.0)		1.00 (0.58–1.71) 0.99	Culled out facilitators with the lowest evaluations	2.01 (1.09–3.71) **0.025 ***	Several of the best facilitators from 2009 were unavailable in 2010	0.52 (0.28 – 0.97) **0.040 ***
	2008	99 / 1	6.4 ± (1.1)	7.0 (1.0)						
	2009	109 / 2	6.7 ± (0.7)	7.0 (0.0)						
	2010	99 / 2	6.5 ± (0.9)	7.0 (1.0)						
5. The facilitators and instructors provided useful feedback	2007	103 / 0	6.4 ± (0.7)	6.0 (1.0)		0.86 (0.52–1.41) 0.54	Culled out facilitators with the lowest evaluations & added dedicated debriefers for all exercises	1.97 (1.20–3.24) **0.008 ***		0.76 (0.45 – 1.27) 0.29
	2008	100 / 0	6.2 ± (1.0)	6.0 (1.0)						
	2009	111 / 0	6.5 ± (0.7)	7.0 (1.0)						
	2010	101 / 0	6.4 ± (0.9)	7.0 (1.0)						
6. I gained more insight into my own strengths and weaknesses as a team member	2007	103 / 0	5.8 ± (1.1)	6.0 (2.0)		1.25 (0.69–2.25) 0.46		1.21 (0.72–2.02) 0.47		1.03 (0.61 – 1.74) 0.90
	2008	100 / 0	5.8 (1.1)	6.0 (2.0)						
	2009	110 / 1	6.0 (1.1)	6.0 (1.0)						
	2010	101 / 0	5.9 (1.2)	6.0 (2.0)						

2

7. I learned to identify the essential behaviors which make a team effective	2007	103 / 0	5.8 ± (0.9)	6.0 (1.0)		0.97 (0.60–1.57) 0.89		1.43 (0.87–2.34) 0.15		1.16 (0.70–1.94) 0.56
	2008	100 / 0	5.7 ± (1.1)	6.0 (1.0)						
	2009	111 / 0	5.9 ± (1.0)	6.0 (2.0)						
	2010	101 / 0	6.0 ± (0.9)	6.0 (2.0)						
8. This course will help me to become a more effective team member of the health care team	2007	101 / 2	5.9 ± (0.9)	6.0 (2.0)		1.25 (0.77–2.03) 0.37		1.53 (0.94–2.50) 0.09		1.07 (0.63–1.84) 0.80
	2008	100 / 0	6.0 ± (1.0)	6.0 (1.0)						
	2009	111 / 0	6.2 ± (0.9)	6.0 (1.0)						
	2010	101 / 0	6.2 ± (1.0)	6.0 (1.0)						
**Teamwork Day Exercise**										
1. *Getting to know who?*	2007	100 / 3	5.3 ± (1.3)	5.0 (1.0)		0.83 (0.52–1.35) 0.46		0.76 (0.47–1.21) 0.24	Replaced by *Name Game* and *Get to Your Spot*	
	2008	97 / 3	5.2 ± (1.3)	5.0 (1.0)						
	2009	110 / 1	4.9 ± (1.5)	5.0 (2.0)						
2. *Name Game*	2010	101 / 0	5.2 ± (1.3)	5.0 (1.0)						
3. *Get to Your Spot*	2010	100 / 1	4.1 ± (1.8)	4.5 (2.0)						
4. *Williams County Medical Center*	2007	100 / 3	5.2 ± (1.4)	5.0 (2.0)	Changed exercise order so this one always preceded	1.72 (1.02–2.89) **0.041 ***		0.76 (0.48–1.20) 0.23		0.76 (0.46–1.27)
	2008	97 / 3	5.8 ± (1.1)	6.0 (2.0)						
	2009	110 / 1	5.5 ± (1.4)	6.0 (1.0)						

3

	2010	101 / 0	5.3 ± (1.5)	6.0 (2.0)	*Ward Rounds*					0.29
5. *Mission to Burundi*	2007	100 / 3	5.4 ± (1.3)	6.0 (1.0)		0.92 (0.56–1.49) 0.72	Replaced by *Pediatric Surgery Scramble*			
	2008	97 / 3	5.3 ± (1.1)	5.0 (1.0)						
6. *Pediatric Surgery Scramble*	2009	110 / 1	5.8 ± (1.2)	6.0 (2.0)					Reduced number of tasks required to be completed	1.29 (0.73 – 2.28) 0.39
	2010	101 / 0	5.8 ± (1.4)	6.0 (2.0)						
7. *Ward Rounds at Jefferson County Medical Center*	2007	100 / 3	6.5 ± (0.8)	7.0 (1.0)		1.05 (0.58 – 1.90) 0.88		0.75 (0.43–1.31) 0.31		1.04 (0.61 – 1.79) 0.88
	2008	97 /3	6.5 ± (0.9)	7.0 (1.0)						
	2009	110 / 1	6.4 ± (0.9)	7.0 (1.0)						
	2010	101 / 0	6.5 ± (0.8)	7.0 (1.0)						
8. *Crisis on Flight 1974*	2007	99 / 4	5.1 ± (1.6)	5.0 (2.0)	Cut team size from 10 to 5 & changed from mannequin to SP	3.92 (2.24–6.86) **<0.001***		1.13 (0.69–1.85) 0.62		0.83 (0.50 – 1.38) 0.47
	2008	97 / 3	6.0 ± (1.1)	6.0 (1.0)						
	2009	109 / 2	6.0 ± (1.3)	6.0 (1.0)						

4

	2010	101 / 0	5.9 ± (1.2)	6.0 (2.0)	passenger (see text for details)					
Colour Blind	2007	100 / 3	6.0 (1.1)	6.0 (1.0)		1.19<br>(0.72-1.98)<br>0.50		1.23<br>(0.71-2.11)<br>0.46		1.44<br>(0.85-2.44)<br>0.18
	2008	97 / 3	6.1 (1.0)	6.0 (2.0)						
	2009	109 / 2	6.2 (1.1)	7.0 (1.0)						
	2010	101 / 0	6.4 (1.0)	7.0 (1.0)						

Evaluation scores ranged from 1 (worst) to 7 (best).

Temporal comparisons of course evaluations. Two ordinal (proportional odds) logistic regression models were estimated, along with their robust standard errors, to characterize the association between scores and the course evaluations and between scores and exercises while adjusting for year and exercise order number. Linear combinations of estimates were calculated to summarize temporal differences.

* = $P<0.05$ indicating statistically significant difference for this comparison

5
