# Supplementary material for: A simulation-based curriculum to introduce key teamwork principles to entering medical students
Source: BMC Med Educ. 2016 Nov 16;16:295. doi: 10.1186/s12909-016-0808-9 (PMC5112730; doi:10.1186/s12909-016-0808-9)
Supplement: Additional file 3: Table S3. — Yearly Comparison of Exercises (P values). (PDF 277 kb) [file 12909_2016_808_MOESM3_ESM.pdf]

Table S3. Yearly Comparison of Exercises (*P values)*

Exercises		Getting to Know Who?	Colour Blind™	Ward Rounds at Jefferson County Medical Center	Crisis on Flight 1974	Williams Medical Center	Mission to Burundi	Pediatric Surgery Scramble	Name Game	Get to your Spot
		OR<br>95% CI<br>P	OR<br>95% CI<br>P	OR<br>95% CI<br>P	OR<br>95% CI<br>P	OR<br>95% CI<br>P	OR<br>95% CI<br>P	OR<br>95% CI<br>P	OR<br>95% CI<br>P	OR<br>95% CI<br>P
Getting to Know Who?	2007	–	***2.75***<br>***(1.71 – 4.43)***<br>***<0.001***	***7.61***<br>***(4.11 – 14.08)***<br>***< 0.001***	0.71<br>(0.37 – 1.36)<br>0.303	0.91<br>(0.49 – 1.66)<br>0.748	0.94<br>(0.50 – 1.74)<br>0.835	–	–	–
	2008	–	***3.92***<br>***(2.36 – 6.51)***<br>***< 0.001***	***9.54***<br>***5.46 – 16.68)***<br>***< 0.001***	***3.33***<br>***(1.93 – 5.76)***<br>***< 0.001***	1.87<br>(1.04 – 3.34)<br>0.036	1.03<br>(0.62 – 1.69)<br>0.916	–	–	–
	2009	–	***6.36***<br>***(3.80 – 10.65)***<br>***< 0.001***	***9.48***<br>***(5.57 – 16.12)***<br>***< 0.001***	***4.98***<br>***(2.84 – 8.75)***<br>***< 0.001***	***1.87***<br>***(1.01 – 3.45)***<br>***0.045***	–	***3.06***<br>***(1.75 – 5.35)***<br>***< 0.001***	–	–

Name Game	2010		***7.06***<br>***(4.32 – 11.52)***<br>***< 0.001***	***7.62***<br>***(4.24 – 13.69)***<br>***< 0.001***	***3.18***<br>***(1.72 – 5.90)***<br>***<0.001***	1.10<br>(0.58 – 2.07)<br>0.776	–	***3.04***<br>***(1.60 – 5.75)***<br>***0.001***	–	***0.31***<br>***(0.19 – 0.52)***<br>***< 0.001***
Get to your Spot	2010	–	***22.66***<br>***(12.92 – 39.75)***<br>***< 0.001***	***24.47***<br>***(12.82 – 46.72)***<br>***< 0.001***	***10.23***<br>***(5.21 – 20.07)***<br>***< 0.001***	***3.52***<br>***(1.77 – 7.02)***<br>***< 0.001***	–	***9.75***<br>***(4.87 – 19.53)***<br>***< 0.001***	***3.21***<br>***(1.91 – 5.39)***<br>***< 0.001***	–
Colour Blind™	2007	***0.36***<br>***(0.23 – 0.59)***<br>***< 0.001***	–	***2.77***<br>***(1.53 – 5.03)***<br>***0.001***	***0.26***<br>***(0.14 – 0.49)***<br>***< 0.001***	***0.33***<br>***(0.18 – 0.59)***<br>***< 0.001***	***0.34***<br>***(0.19 – 0.62)***<br>***< 0.001***	–	–	–
	2008	***0.25***<br>***(0.15 – 0.42)***<br>***< 0.001***	–	***2.43***<br>***(1.34 – 4.42)***<br>***0.004***	0.85<br>(0.47 – 1.53)<br>0.588	***0.48***<br>***(0.25 – 0.89)***<br>***0.020***	***0.26***<br>***(0.15 – 0.45)***<br>***< 0.001***	–	–	–
	2009	***0.16***<br>***(0.09 – 0.26)***<br>***< 0.001***	–	1.49<br>(0.86 – 2.59)<br>0.157	0.78<br>(0.44 – 1.41)<br>0.414	***0.29***<br>***(0.16 – 0.56)***<br>***< 0.001***	–	***0.48***<br>***(0.27 – 0.86)***<br>***0.014***	–	–
	2010	–	–	1.08<br>(0.58 – 1.99)<br>0.806	***0.45***<br>***(0.24 – 0.86)***<br>***0.015***	***0.16***<br>***(0.08 – 0.30)***<br>***< 0.001***	–	***0.43***<br>***(0.22 – 0.84)***<br>***0.013***	***0.14***<br>***(0.09 – 0.23)***<br>***< 0.001***	***0.04***<br>***(0.03 – 0.08)***<br>***< 0.001***

Ward Rounds at Jefferson County Medical Center	2007	***0.13<br>(0.07 – 0.24)<br>< 0.001***	***0.36<br>(0.20 – 0.66)<br>0.001***	–	***0.09<br>(0.05 – 0.16)<br>< 0.001***	***0.12<br>(0.07 – 0.20)<br>< 0.001***	***0.12<br>(0.07 – 0.21)<br>< 0.001***	–	–	–
	2008	***0.10<br>(0.06 – 0.18)<br>< 0.001***	***0.41<br>(0.23 – 0.75)<br>0.004***	–	***0.35<br>(0.20 – 0.62)<br>< 0.001***	***0.20<br>(0.11 – 0.36)<br>< 0.001***	***0.11<br>(0.06 – 0.18)<br>< 0.001***	–	–	–
	2009	***0.11<br>(0.06 – 0.18)<br>< 0.001***	0.67<br>(0.39 – 1.17)<br>0.157	–	***0.53<br>(0.31 – 0.89)<br>0.016***	***0.20<br>(0.11 – 0.35)<br>< 0.001***	–	***0.32<br>(0.19 – 0.54)<br>< 0.001***	–	–
	2010	–	0.93<br>(0.50 – 1.71)<br>0.806	–	***0.42<br>(0.24 – 0.72)<br>0.002***	***0.14<br>(0.08 – 0.26)<br>< 0.001***	–	***0.40<br>(0.23 – 0.70)<br>0.001***	***0.13<br>(0.07 – 0.24)<br>< 0.001***	***0.04<br>(0.02 – 0.08)<br>< 0.001***
Crisis on Flight 1974	2007	1.41<br>(0.73 – 2.70)<br>0.303	***3.87<br>(2.05 – 7.31)<br>< 0.001***	***10.71<br>(6.18 – 18.56)<br>< 0.001***	–	1.28<br>(0.73 – 2.24)<br>0.397	1.32<br>(0.76 – 2.28)<br>0.323	–	–	–
	2008	***0.30<br>(0.71- 0.52)<br>< 0.001***	1.18<br>(0.65 – 2.12)<br>0.588	***2.86<br>(1.61 – 5.08)<br>< 0.001***	–	***0.56<br>(0.34 – 0.92)<br>0.021***	***0.31<br>(0.19 – 0.49)<br>< 0.001***	–	–	–

	2009	***0.20***<br>***(0.11 – 0.35)***<br>***< 0.001***	1.28<br>(0.71 – 2.29)<br>0.414	***1.90***<br>***(1.13 – 3.21)***<br>***0.016***	–	***0.38***<br>***(0.23 – 0.61)***<br>***< 0.001***	–	***0.61***<br>***(0.37 – 1.01)***<br>***0.054***	–	–
	2010	–	***2.22***<br>***(1.17 – 4.21)***<br>***0.015***	***2.39***<br>***(1.38 – 4.14)***<br>***0.002***	–	***0.34***<br>***(0.19 – 0.61)***<br>***< 0.001***	–	0.95<br>(0.53 – 1.71)<br>0.872	***0.31***<br>***(0.71 – 0.58)***<br>***< 0.001***	***0.10***<br>***(0.05 – 0.19)***<br>***< 0.001***
Williams Medical Center	2007	1.10<br>(0.60 – 2.02)<br>0.748	***3.03***<br>***(1.68 – 5.47)***<br>***< 0.001***	***8.40***<br>***(4.99 – 14.15)***<br>***< 0.001***	0.78<br>(0.45 – 1.38)<br>0.397	–	1.03<br>(0.60 – 1.77)<br>0.904	–	–	–
	2008	***0.54***<br>***(0.30 – 0.96)***<br>***0.036***	***2.10***<br>***(1.12 – 3.94)***<br>***0.020***	***5.12***<br>***(2.81 – 9.31)***<br>***< 0.001***	***1.79***<br>***(1.09 – 2.93)***<br>***0.021***	–	0.55<br>(0.35 – 0.88)<br>0.12	–	–	–
	2009	***0.53***<br>***(0.29 – 0.99)***<br>***0.045***	***3.40***<br>***(1.80 – 6.42)***<br>***< 0.001***	***5.07***<br>***(2.87 – 8.93)***<br>***< 0.001***	***2.66***<br>***(1.64 – 4.33)***<br>***< 0.001***	–	–	1.64<br>(0.97 – 2.77)<br>0.066	–	–
	2010		***6.44***<br>***(3.30 – 12.54)***<br>***< 0.001***	***6.95***<br>***(3.87 – 12.49)***<br>***< 0.001***	***2.90***<br>***(1.64 – 5.15)***<br>***< 0.001***	–	–	***2.77***<br>***(1.50 – 5.10)***<br>***0.001***	0.91<br>(0.48 – 1.72)<br>0.776	***0.28***<br>***(0.14 – 0.57)***<br>***< 0.001***

Mission to Burundi	2007	1.07<br>(0.57 – 1.99)<br>0.835	***2.93***<br>***(1.61 – 5.35)***<br>***< 0.001***	***8.13***<br>***(4.86 – 13.58)***<br>***< 0.001***	0.76<br>(0.44 – 1.31)<br>0.323	0.97<br>(0.56 – 1.66)<br>0.904	–	–	–	–
	2008	0.97<br>(0.59 – 1.60)<br>0.916	***3.82***<br>***(2.21 – 6.60)***<br>***< 0.001***	***9.29***<br>***(5.48 – 15.76)***<br>***< 0.001***	***3.25***<br>***(2.03 – 5.19)***<br>***< 0.001***	***1.82***<br>***(1.14 – 2.89)***<br>***0.012***	–	–	–	–
Pediatric Surgery Scramble	2009	***0.33***<br>***(0.19 – 0.57)***<br>***< 0.001***	***2.08***<br>***(1.16 – 3.73)***<br>***0.014***	***3.10***<br>***(1.85 – 5.17)***<br>***< 0.001***	***1.63***<br>***(0.99 – 2.67)***<br>***0.054***	0.61<br>(0.36 – 1.03)<br>0.066	–	–	–	–
	2010	–	***2.32***<br>***(1.20 – 4.52)***<br>***0.013***	***2.51***<br>***(1.42 – 4.43)***<br>***0.001***	1.05<br>(0.59 – 1.88)<br>0.872	***0.36***<br>***(0.20 – 0.66)***<br>***0.001***	–	–	***0.33***<br>***(0.17 – 0.62)***<br>***0.001***	***0.10***<br>***(0.05 – 0.21)***<br>***< 0.001***

Yearly exercise comparisons. An ordinal (proportional odds) logistic regression model was estimated, along with its robust standard errors, to characterize the association between scores and exercises while adjusting for year and exercise order. Linear combinations of estimates were calculated to summarize exercise differences by year. The scores that were statistically significant have been italicized in the table.
